# Supplementary material for: Medical students impacted by discrimination: a qualitative study into their experiences of belonging and support systems at medical schools in the UK
Source: BMJ Open. 2023 Dec 28;13(12):e078314. doi: 10.1136/bmjopen-2023-078314 (PMC10759082; doi:10.1136/bmjopen-2023-078314)
Supplement: Supplementary data [file bmjopen-2023-078314supp001.pdf]

APPENDICES

Appendix 1 - Interview Questions

1. Warm up: Can you tell me how you have generally found studying medicine? Is there any particular reason why you chose this medical school?
Prompt: Confidence with learning, and workload levels?

2. Have you had any experiences during your time at medical school that made you feel as though you belonged?

Prompt: Could you tell me a little bit more about that? What did you learn from that experience? How has this experience impacted you going forward?

3. Have you had any experiences during your time at medical school that made you feel as though you didn't belong?
Prompt: Could you tell me a little bit more about that? What did you learn from that experience? How has this experience impacted you going forward?

4. Have you found anything to be particularly challenging or difficult as a medical student impacted by discrimination?
Prompt: Can you tell me a bit more about that? What did you learn from that experience? Will/how will that change how you see things or act moving forwards?

5. What support currently exists for you at medical school? Have you accessed any of these? Has this proved satisfactory/useful?
Prompt: Are there any structures in particular? How many of these systems are student-led?

6. What support currently exists for you outside of the formal support provided by your medical school?
Prompt: Does the support within medical school or these wider networks foster a greater sense of belonging?

7. At the moment, how are your working relationships within your medical school? (Rephrase if not understood - How do you feel like your professional relationships with your peers are? And then can ask about medical school staff).
Prompt: And what about your peers? What about with staff at the medical school?

8. How are your working relationships on clinical placements? (Rephrase if not understood - How do you feel like your professional relationships with the staff on placements are?)

Prompt: With doctors? And with patients?

— TO ASK TO ROUND UP Q8: Do you feel like you belong in the clinical environment?

9. At the moment, are there any areas where you feel you could do with more support/supervision to improve your sense of belonging?
Prompt: Do you feel there is anything lacking in terms of support at your medical school? And in what areas do you feel you are represented in the curriculum? Can this be improved?

10. At the moment, how has the experience of being discriminated against impacted your overall feelings regarding your studies at medical school?
Prompt: How has this impacted your sense of belonging in particular?
Has anything changed for you over your time at university in terms of feeling a sense of belonging?
How has this impacted your feelings about becoming a doctor and being a part of the medical profession?

11. Is there anything else you'd like to share about your experiences of belonging and support at medical school?
Prompt: Is there anything you feel we haven't covered in this interview that we should have?

12. Do you have any questions that you would like to ask?

Debriefing information (see welfare support sheet)

Appendix 2 - Recruitment Text

Medical students impacted by discrimination: Their experiences of sense of belonging and support systems at medical schools in the United Kingdom.

With so many initiatives to improve the diversity of medical schools, we're a team of UK medical students interested in finding out just how much minoritized medical students feel they belong and are supported past the application process of their respective schools. We are interested in hearing your honest experiences in a bid to help shape medical education in the UK.

To participate in this study you must have attended or are currently attending the following medical schools:

- Imperial College London
- University College London
- University of Dundee
- University of Nottingham Schools (Nottingham and Lincoln Campus)

For the purposes of this research, we have defined minoritized as someone who has felt that they have been impacted by discrimination. Examples of minoritized groups include (but are not limited to) the following:

- Those who identify as being otherly-abled/ having a disability/ Receiving DSA
- Those who belong to an ethnic minority
- Those who identify as LGBTQIA+

- First generation applicants to higher education
- Those previously looked after by a local authority
- Those who are themselves, or have parents/ guardians in receipt of means-tested benefits
- Household addresses in a POLAR 3 or 4 area
- Those with refugee status
- A young carer
- Students who belong to one of the priority engagements groups (include students from Gypsy, Roma and Traveller communities and children on military families)
- Those who are estranged from both parents/ legal guardians during their higher education
- Mature students

All students who apply and are selected to participate in our interviews, which should take no longer than 60 minutes, will receive a £20 shopping voucher at the end of the process as compensation for their time.

If you are interested in taking part, please click the link to the following survey to register your interest:

tinyurl.com/ICLSEDFORM

If you have any questions regarding this project, please contact: Hamza Ikhlaq via email: hi418@ic.ac.uk
