# Supplementary material for: Medical students impacted by discrimination: a qualitative study into their experiences of belonging and support systems at medical schools in the UK
Source: BMJ Open. 2023 Dec 28;13(12):e078314. doi: 10.1136/bmjopen-2023-078314 (PMC10759082; doi:10.1136/bmjopen-2023-078314)
Supplement: Author's manuscript [file bmjopen-2023-078314.draft_revisions.pdf]

BMJ Open

BMJ Open is committed to open peer review. As part of this commitment we make the peer review history of every article we publish publicly available.

When an article is published we post the peer reviewers' comments and the authors' responses online. We also post the versions of the paper that were used during peer review. These are the versions that the peer review comments apply to.

The versions of the paper that follow are the versions that were submitted during the peer review process. They are not the versions of record or the final published versions. They should not be cited or distributed as the published version of this manuscript.

BMJ Open is an open access journal and the full, final, typeset and author-corrected version of record of the manuscript is available on our site with no access controls, subscription charges or pay-per-view fees (http://bmjopen.bmj.com).

If you have any questions on BMJ Open's open peer review process please email info.bmjopen@bmj.com

BMJ Open

Medical students impacted by discrimination: a qualitative study into their experiences of belonging and support systems at medical school

Journal:	*BMJ Open*
Manuscript ID	bmjopen-2023-078314
Article Type:	Original research
Date Submitted by the Author:	29-Jul-2023

Complete List of Authors:	Ikhlaq, Hamza; Imperial College London, Faculty of Medicine<br>Agarwal, Srishti; University College London<br>Kwok, Catharine; University College London<br>Golamgouse, Hadiya; University College London<br>Derby, Simone ; University of Nottingham<br>McRae, Nicola; University of Dundee<br>Brown, Megan; Imperial College London, Medical Education Innovation and Research Centre (MEdIC)<br>Collin, Victoria; Imperial College London, Medical Education Innovation and Research Centre (MEdIC)<br>Parekh, Ravi; Imperial College London, Medical Education Innovation & Research Centre, School of Public Health<br>Kumar, Sonia; University of Leeds

Keywords:	MEDICAL EDUCATION & TRAINING, QUALITATIVE RESEARCH, Social Support

SCHOLARONE™
Manuscripts

I, the Submitting Author has the right to grant and does grant on behalf of all authors of the Work (as defined in the below author licence), an exclusive licence and/or a non-exclusive licence for contributions from authors who are: i) UK Crown employees; ii) where BMJ has agreed a CC-BY licence shall apply, and/or iii) in accordance with the terms applicable for US Federal Government officers or employees acting as part of their official duties; on a worldwide, perpetual, irrevocable, royalty-free basis to BMJ Publishing Group Ltd ("BMJ") its licensees and where the relevant Journal is co-owned by BMJ to the co-owners of the Journal, to publish the Work in this journal and any other BMJ products and to exploit all rights, as set out in our licence.

The Submitting Author accepts and understands that any supply made under these terms is made by BMJ to the Submitting Author unless you are acting as an employee on behalf of your employer or a postgraduate student of an affiliated institution which is paying any applicable article publishing charge ("APC") for Open Access articles. Where the Submitting Author wishes to make the Work available on an Open Access basis (and intends to pay the relevant APC), the terms of reuse of such Open Access shall be governed by a Creative Commons licence – details of these licences and which Creative Commons licence will apply to this Work are set out in our licence referred to above.

Other than as permitted in any relevant BMJ Author's Self Archiving Policies, I confirm this Work has not been accepted for publication elsewhere, is not being considered for publication elsewhere and does not duplicate material already published. I confirm all authors consent to publication of this Work and authorise the granting of this licence.

For peer review only

H Ikhlaq, S Agarwal, C Kwok, H Golamgouse, S Derby, N McRae
M Brown, V Collin, R Parekh, S Kumar
Imperial College London, The Reynolds Building
St Dunstans Road, London
W6 8RP, UK
17th May 2023

Adrian Aldcroft
Editor
BMJ Open

Dear Mr Aldcroft,

We are writing to submit our manuscript entitled, '*Medical students impacted by discrimination: a qualitative study into their experiences of belonging and support systems at medical school.'* for consideration as a BMJ Open article.

We conducted a qualitative study to analyse the experiences of medical students impacted by discrimination. Existing research in this field tends to focus on one particular group, therefore we believe our findings will better reflect the sense of belonging felt by medical students impacted by discrimination. Additionally, we provide recommendations for formal and informal provisions to foster a greater sense of belonging for such students.

This project was competitively chosen as a result of the Imperial College Medical Education Innovation and Research Centre (MEdIC) masterclasses. This multi-institutional project involved a group collaboration between medical student researchers and supervising academics from MEdIC to investigate student experiences and produce the referenced manuscript.

We declare no ongoing conflicts of interest associated with our submission. H Ikhlaq, S Agarwal, C Kwok, H Golamgouse, S Derby and N McRae contributed to the study conception, protocol development, data collection, data interpretation and critical revision of the manuscript. M Brown, V Collin, R Parekh and S Kumar provided overall supervision and support utilising their expertise in medical education.

We confirm that this manuscript has not been published elsewhere and is not currently being considered by another journal.. All authors have approved this edition and agree with the submission to BMJ Open.

Thank you very much in advance for taking the time to read our manuscript. We greatly appreciate your feedback and look forward to reviewing your comments.

Kind regards,

For peer review only

Hamza Ikhlaq, Srishti Agarwal, Catharine Kwok, Hadiya Golamgouse, Simone Derby, Nicola McRae, Dr Megan Brown, Dr Victoria Collin, Dr Ravi Parekh, Professor Sonia Kumar

For peer review only

Medical students impacted by discrimination: a qualitative study into their experiences of belonging and support systems at medical school

Hamza Ikhlaq[1], Srishti Agarwal[2], Catharine Kwok[2], Hadiya Golamgouse[2], Simone Derby[3], Nicola McRae[4], Dr Megan Brown[1], Dr Victoria Collin[1], Dr Ravi Parekh[1] and Professor Sonia Kumar[5]

[1]Imperial College London, The Reynolds Building,
St Dunstans Road, London, W6 8RP

[2]University College London,
74 Huntly Street, London, WC1E 6BT

[3]University of Nottingham Medical Schools
East Block, Lenton, Nottingham NG7 2UH

[4]University of Dundee,
Nethergate, Dundee, DD1 4HN

[5]University of Leeds,
Faculty of Medicine and Health, Leeds, LS2 9JT

Correspondence concerning this article should be addressed to Hamza Ikhlaq
Imperial College London, The Reynolds Building
St Dunstans Road, London, W6 8RP
Email: hamza.ikhlaq18@imperial.ac.uk

Abstract: 197 words
Manuscript: 4215 words

For peer review only

ABSTRACT

Objective: To better understand the broader experience of medical students impacted by discrimination and the support systems they engage with.

Design: Qualitative study using semi-structured interviews.

Setting: Four medical schools based in the United Kingdom.

Participants: 17 medical students were recruited using volunteer and snowball sampling: all students self-identified as being impacted by discrimination.

Results: 5 themes were identified: feelings of isolation, imposter syndrome and exclusion; a lack of representation and positive role modelling; the importance of peer support; issues relating to the accessibility of support; building support networks through shared experiences and attempts to foster a sense of inclusion through peer and institutionally led initiatives.

Conclusions: The findings of this study suggest medical schools are failing to recognise the importance of acknowledging the multiple identities at risk of discrimination held by students, perpetuating feelings of isolation and exclusion. Our research highlights the need for practical systemic initiatives to improve the sense of belonging of medical students who are impacted by discrimination. Medical educators and institutions should consider formal and informal provisions, such as creating time and space for students to meet and share experiences, access support and reporting networks, to foster a greater sense of belonging.

Strengths and limitations of this study

- This study utilised a novel method of exploring peer perspectives via a team largely composed of medical student researchers, whom themselves have lived experiences of discrimination within medical school.
- Our research is unique in its approach as it examined student belonging from a broader perspective, hearing from those impacted by discrimination, and acknowledging the multiple identities that students can have.
- Although this study has a small sample, it captures the range of experiences across four institutions and across multiple years of medical school programmes.
- Given the complexity and sensitivity of discrimination in medical school, semi-structured interviews have allowed us to explore the personal and often sensitive experiences of participants.
- This multi-institutional study covers 4 of the 46 current medical schools in the UK. Therefore, there is scope for further examining the experiences of students with intersecting identities and experiences across varying geographical locations within the UK and beyond.

Funding statement

This article presents independent research commissioned by the National Institute for Health Research (NIHR) under the Applied Health Research (ARC) programme for North West London. The views expressed in this publication are those of the author(s) and not necessarily those of the NHS, the NIHR or the Department of Health and Social Care.

Keywords

discrimination, belonging, medical, student, support

INTRODUCTION

To experience discrimination is to be treated unfairly or with prejudice based on the characteristics of a group, or an individual (1). Terrell L. Strayhorn (2) defined a sense of belonging within higher education as:

> 'students' perceived social support on campus, a feeling or sensation of connectedness, and the experience of mattering or feeling cared about, accepted, respected, valued by, and important to the campus community or others on campus such as faculty, staff, and peers.' (p.4).

Belonging has been linked to positive academic outcomes, high engagement and self-confidence amongst university students (3) and is pertinent in students who are impacted by discrimination as they are already susceptible to exclusion and isolation (4). Such students who have reported mistreatment and discrimination in medical school, particularly those in their earlier years, have subsequently been found to be more likely to discontinue from medical school (5).

Historically, the makeup of medical students in the UK is skewed towards students from more affluent backgrounds (6). As a result, UK medical schools are now obliged to have schemes that ensure medicine is accessible to a wider population (7). Widening participation (WP) is an example of such an approach and is defined as: 'ensuring that students from disadvantaged backgrounds can access higher education, get the support they need to succeed in their studies, and progress to further study and/or employment suited to their qualifications and potential' (6). However, students impacted by discrimination , whether it be due to their WP status or those at risk of societal discrimination due to minoritised characteristics, have been found to face barriers in every stage of their medical careers (8).

According to Jackson, 'Fairness and equality in medical education is not just a problem at the front door; it continues throughout medical school.' (9) Current literature primarily focuses on interventions for post-16 students to boost enrolment into medical school. However, there is limited research into the follow up of students impacted by discrimination during their time in medical school (10). There is emerging evidence that students facing discrimination have different learning and social experiences and further research is required to address systemic issues within medical schools (11). Indeed, the prescriptive nature of simply increasing the

For peer review only - http://bmjopen.bmj.com/site/about/guidelines.xhtml

number of students from disadvantaged backgrounds into medical schools has a risk of 'tokenising' these students, and further alienating their sense of belonging (12). Medical students subject to discrimination can experience social exclusion from the wider cohort and inequality in accessing resources. This can result in a diminished sense of trust between these students and the institution (13). There are also specific barriers such as fears of stigmatisation and a lack of accessibility for neurodivergent students (14, 15), which influences a poorer sense of belonging due to poor relationships with the institution, staff, and clinicians (13). Though important, and interesting, these studies are limited in that they typically focus on exploring one group of student's sense of belonging such as ethnically minoritised students, or socio-economically disadvantaged populations.

There is a need for further research to examine the wider experiences of medical students impacted by discrimination and the intersections of identities for students who may have two or more characteristics that are at risk of discrimination. As such, their experiences of belonging may be significantly different from those previously researched. Current literature provides narrative accounts of the experiences of students who experience discrimination and identifies factors which drive discrimination. However, there is limited data on what support is available for these students and if available, how effective this may be. Furthermore, it is imperative to understand what support these students would require from their universities to maximise student engagement.

A collective group of medical students from four UK-based medical schools, with a shared concern in this area, met through a series of medical education masterclasses run by Imperial College London in the summer of 2021. As a result, this student-led project examined the experiences of medical students who have been impacted by discrimination, and the support systems available to them. We aim to generate practical recommendations to improve systemic and holistic provisions for these students thereby enhancing their sense of belonging.

METHODS

Given the complexity and sensitivity of discrimination in medical school, we chose to conduct semi-structured interviews to explore the thoughts, beliefs, and experiences of participants. We created 12 questions to form the basis of our semi-structured interviews covering various themes of belonging, networks, support systems and experiences of discrimination using findings from our literature review. Each participant was interviewed individually (Appendix 1). We used a constructivist paradigm (16) using an interpretative description approach (17). This enabled us to fully explore each participant's subjective experiences and understand the influences behind these, in order to develop practical recommendations to improve belonging and support systems at medical school. Data were collected between October to December 2022; ethical approval was given by the Educational Ethics Research Committee at **Imperial College London,** with reciprocal approval granted by partner medical schools of the research team (reference number: EERP2122-068a). All participants were emailed welfare debriefing information specific to their institution and SK was the assigned point of contact in the event of serious concern.

Sample and Recruitment

Medical students who self-identified as having been impacted by discrimination were eligible to take part in the study. Students who had just undertaken an intercalated BSc were excluded from the study, as they may be affiliated to a department external to the medical school and not all medical students may have the opportunity to intercalate. Participants self-identified as having been impacted by discrimination using an existing, broadened widening participation definition (18). This allowed us to collect a diverse multitude of student experiences, which may be currently underrepresented in existing literature and policies. Our recruitment text provided some guidance through examples of discriminated groups including but not limited to:

- Those who identify as being otherly-abled/having a disability/receiving DSA
- Those who belong to an ethnic minority
- Those who identify as LGBTQIA+
- First generation applicants to higher education
- Those previously looked after by a local authority
- Those who are themselves, or have parents/guardians in receipt of means-tested bene benefits
- Household addresses in a POLAR 3 or 4 area
- Those with refugee status
- A young carer
- Students who belong to one of the priority engagements groups (include students from Gypsy, Roma and Traveller communities and children on military families)
- Those who are estranged from both parents/legal guardians during their higher education
- Mature students

For the full recruitment text, see Appendix 2.

We recruited through the host institution using email, newsletters, and relevant social media platforms. Adverts were sent out through these platforms asking students to sign up. These students were then contacted by the research team to check eligibility and availability for an interview.

In addition to fulfilling our recruitment criteria, participants were eligible to participate if they were available for an interview and provided consent to participate. We aimed to ensure breadth of experiences, maximising variability across year groups; for each university, we selected one student per year group. All participants were compensated for their time through £20 food vouchers. In total, we recruited 17 participants from all years of the medical school

For peer review only

programmes across four UK-based medical schools using convenience sampling. This allowed us to achieve our study aims and ensure a breadth of experiences, exploring their experiences to draw conclusions regarding their sense of belonging and experience of support at medical school.

Procedure

After selection, each participant was invited to a 60-minute semi-structured interview. Each interview was run by two co-investigators (HI, SA, CK, HG, NM, SD), with one leading and one supporting the interview (prompting additional questions to explore students' experiences or in case of technical difficulties). Consent was gained prior to the interview through submission of an online consent form and verbal consent to record the session was obtained prior to the start of each discussion. To avoid potential conflicts of interest, investigators only interviewed participants external to their own institutions.

Data Analysis

After transcription, we anonymised data and followed a constructivist approach to qualitative thematic analysis. We analysed data inductively and followed Braun and Clarke's (16) six steps: familiarisation; data coding; generating initial themes; reviewing and developing themes; refining, defining and naming themes; and writing the report. We developed a code list, which all members of our team reviewed. At least one member of our team peer-reviewed each transcript to add depth to coding (HI, SA, CK, HG, NM, SD). Throughout the analysis, we modified existing codes and created new codes through discussion. We then connected these codes to develop themes through team discussions. All data analysis was conducted using the Dedoose (v9.0) software.

Patient and public involvement

Patients or the public were not involved in the design or conduct of this study.

Reflexivity

The research team come from varying ethnically minoritised backgrounds and is predominantly female identifying, all with a strong interest in medical education. All student researchers identify as having been impacted by discrimination, thus leading the research team to explore this research project and have a strong aspiration to make positive recommendations for medical schools. Due to the nature of our study and personal experiences of discrimination, peer debriefing through online meetings was used to reflect and deepen our understanding of recounted experiences shared by study participants and provide support if sensitive topics arose.

RESULTS

For peer review only

Five themes were identified from the 17 interviews conducted: feelings of exclusion; failures of organised support systems; representation within the medical school curriculum, staff and peers; the importance of peer support and attempts to foster a sense of inclusion. A summary of the characteristics of our study population is provided below (Table 1).

Table 1 Characteristics of our Study Population

Participant	Gender	Medical School	Year Group in the academic year 2021-2022
P001	Male	Imperial College London	1
P002	Non-Binary	Imperial College London	2
P003	Male	Imperial College London	3
P004	Non-Binary	Imperial College London	4
P005	Female	Imperial College London	5
P006	Female	University College London	1
P007	Female	University College London	2
P008	Male	University College London	3
P008	Female	University College London	4
P009	Male	University of Nottingham and Lincoln Medical Schools	1
P010	Female	University of Nottingham and Lincoln Medical Schools	2
P011	Female	University of Nottingham and Lincoln Medical Schools	3
P012	Female	University of Nottingham and Lincoln Medical Schools	4
P013	Female	University of Dundee	1
P015	Female	University of Dundee	2
P016	Female	University of Dundee	3
P017	Female	University of Dundee	4

For peer review only

Feelings of exclusion

One of the main barriers to achieving a sense of belonging experienced by participants was feelings of exclusion at an individual and institutional level. At an individual level, minoritised characteristics such as ethnicity, socioeconomic class, religious beliefs, and caring responsibilities were key factors which participants felt perpetuated feelings of exclusion. For example, one participant highlighted the lack of diversity amongst peers as the reason they 'never feel' like they 'fully belong' (P012). This was further exacerbated by intersections of identity as 'being an ethnic minority wearing a hijab' made them feel they were 'a minority within a minority' (P012). Opportunities to socialise with peers sometimes conflicted with participants' cultural and religious beliefs where 'every single event centred around drinking' with a lack of alternative events available (P005). Furthermore, participants highlighted when feelings of exclusion were felt it was because they 'couldn't connect' to their peers due to their unique experiences such as 'experiences as a care leaver', thus resulting in self 'blame' to explain the 'disconnect' (P002).

At an institutional level, participants expressed a disconnect between the medical schools and students. One student felt that they felt they were 'set up to fail' in a system which does not recognise their intersection of identities, thus 'disempowering' them (P008). Feelings of exclusion echoed into clinical attachments where students felt senior doctors would make 'split-second' judgements based on 'prejudice', to the extent they felt 'belittled' (P012). Examples of this emerged particularly in surgical theatres where students who wore hijab felt 'discouraged from going to theatre' (P012).

Failures of Support Systems

Participants had experienced failings in the formal support networks and structures provided by their medical schools. Fixed structures, such as requiring a tutor referral for welfare support made it 'very difficult… to access other welfare support services' (P002) when their tutor had been uncontactable for an extended period of time. This barrier to accessing pastoral support was further demonstrated by another participant questioning: 'how desperate are you to go through all… those hoops to actually get the support?' (P004).

Other failings have included experiences of being 'bounced between people and no one really is quite sure what's happening' (P008). Consequently, this 'one size fits all' (P003) approach has built a feeling of lack of genuine care: 'they want to tick a box saying they've provided support' (P012) and one participant expressed that they had 'a level of distrust …within the system' (P008). Lack of follow-up and ongoing support was evidenced: 'it almost feels prescriptive, that's just like okay, we've met, great, we've done our job, you must be better now' (P004).

Representation

Representation in medical school was a common topic that was raised as a source of comfort, helping to improve belonging, and equally, the lack thereof challenged participants.

Having an open space to discuss identities, alongside others similarly identifying individuals helped provide a sense of familiarity to participants, namely for LGBTQ+ individuals who struggled to find this in the 'heteronormative blanket' (P008). Additionally, the diversity of

For peer review only

faculty members also greatly helps to improve belonging, with one participant 'so genuinely happy to see a hijabi lecturer because it was the only one I've seen' (P012).

Issues with representation arose on several occasions, such as the integral makeup of students with 'most' of the student population being educated in 'private schools' and the class divide that inherently exists between these students and students impacted by discrimination (P012). Furthermore, participants felt they may be represented in faculty as a 'woman' but did not feel the same with regards to their 'ethnicity'. (P013) Additionally, the lack of inclusive teaching, including signs and skin pathology in ethnic minority patients, were raised twice (P012, P013) as a significant problem that impacts belonging. Indeed, the '(lack of teaching) makes you feel like *you* don't matter, *you're not important*.' (P012)

Despite this, one student did raise some improvements that have been made as a whole, which has improved their sense of belonging through representation. There has been more 'active efforts to embrace people of different backgrounds' in clinical and academic environments which have been 'successful' and have increased their sense of belonging (P003).

Peer Support

Some participants described how peer support, resulting from positive interpersonal relationships between themselves and members of their cohort, and the wider university population have impacted their experiences at medical school as students who have experienced discrimination. For those who have very little or no peer support, there was a desire to have that access. Having friends to help provide support was said to be integral in providing 'emotional padding' (P002) which some students have found was often omitted from their lives up to this point. To some students, the willingness of others to talk 'openly and give support' is a 'big deal' (P003), especially for those who are going through what seems to be challenging times.

However, participants specified preferences that would help maximise the efficiency of peers as a support system which included peers who are 'of older years' P002 and/ or with whom they 'have that shared experience' (P001). This stems from the notion that as much as 'peers who aren't from that background try to empathise and try to relate…they don't have the lived experience' (P003) which creates a barrier in feeling understood and supported.

Feelings of Inclusion

Participants tended to express feelings of inclusion when reflecting on their experiences with communities that they 'had a lot in common with' (P002). The freedom to be who you are without judgement was important to most students who found comfort in diverse student groups ranging from sports to LGTBQ+ societies. Feeling like 'something bigger than yourself' gave students the strength to 'be sort of whatever you want to be' (P003) and which in turn improved their overall sense of belonging.

However, feeling included was a process that required active effort 'I've had to fight to think that I belong' (P003). Several participants changed elements of themselves to better fit in, which only added to feeling more out of place. Feelings of inclusion were also constantly

For peer review only

challenged; by themselves, by their peers, and by the medical school throughout their time at university. Imposter syndrome was attributed to this challenge. Students struggled to keep up with their peers and thought they weren't ''smart enough' (P008) to be at medical school. The lack of support at this level meant that students held onto these beliefs until they found a community which gave them 'the confidence to do well, but also equally fail' (P003).

Additionally, several students considered loneliness when reflecting on their experiences of inclusion. Many described feeling isolated from their peers due to characteristics largely outside of their control such as gender, and sexuality. Despite overcoming several hurdles to reach medical school, participants described feeling as if they alone did not belong and that they were out of place. As stated by P002 'For me, I took most of my first year of medical school thinking 'oh my god, I'm the only one' that everyone else was normal'. The importance of community was therefore highlighted in all participant reflections and was explored as a primary cause for improved feelings of inclusion.

DISCUSSION

Through semi-structured interviews, we explored the perceived sense of belonging felt by medical students across four UK medical schools who have been impacted by discrimination and the support systems they have accessed. Our study highlights how feelings of exclusion prevent medical students from achieving a sense of belonging. Students from minoritised backgrounds reported a lack of peer diversity and understanding of their experiences from peers. Furthermore, barriers to engaging in accessible social and professional activities often impacted participants' self-esteem.

Diversity in student makeup has been found to be associated with perceived enhancements in educational experiences (19). Furthermore, works by Machado et al. have suggested socioeconomic and racial diversity offers opportunities to reform medical culture, but student socialisation must be supported through activities to support self-esteem (20). Students also described experiences of marginalisation and stereotyping in clinical attachments, exacerbated by the professional hierarchy, which has been linked to moral distress, trainee mistreatment and amplification of fatigue, stress, and burnout (21). As well as this being reflected in our study, we find feelings of self-blame and a resultant lack of self-worth sometimes are used by students to explain the disconnect between their experiences and those of their non-marginalised peers, further disempowering them from fully engaging with clinical, non-clinical and social experiences provided within the medical school.

The need for representation extends within the formal curricula as participants highlighted the lack of inclusive teaching on disease in ethnically minoritised patients. Considering the topics discussed above, it is perhaps not surprising that participants felt displaced when teaching solely focused on white skin, with signs and symptoms only noted in those belonging to said populations (22). Participants in this study highlighted how seeing themselves represented within the curriculum allowed them to feel a greater sense of self-importance and observing active institutional efforts to diversify the curriculum fostered positive feelings towards their respective medical schools. Thus, representation, both in the physical form of a diverse and intersectional cohort of staff and doctors, coupled with inclusive, non-stereotyped, teaching, and the provision of allocated spaces to discuss these pertinent issues would greatly improve the sense of belonging for students impacted by discrimination.

For peer review only

Students reported experiences of barriers to accessing support through often hard-to-reach, unavailable networks, or outdated systems. The ease of access is a key area for institutions to focus on, for example, by providing multiple access points (23). Institutions should be proactively encouraging students to seek support at an earlier and more manageable stage, ensuring that follow-up opportunities are integrated. We have demonstrated that when a 'one size fits all' and tokenistic system of support disappoints an individual, students have felt isolated and cynical. Providing individualised support tailored to appropriately match the diverse identities of the student population is paramount in order to bridge this disconnect between stated and perceived institutional values (24). We have demonstrated this is vital in ensuring students can build authentic connections with support staff to establish trust and reduce the confusion felt by students when attempting to access relevant support services. However, to implement such support, pastoral support needs to be prioritised and appropriately renumerated to overcome logistical barriers, such as time and financial costs.

This research has shown that across cohorts, medical students who have experienced discrimination desire peer-to-peer support, to help improve their sense of belonging. However, this peer support must be done in a way in which students feel will be most beneficial, such as from older students, those considered friends and those with a shared experience as them. Peer-to-peer support has been shown to help medical students who may feel burnt out, inadequate, socially isolated, and anxious (25), and is found to have positive impacts on relationships with peers as they become professionals (26). The development of peer support systems that are established in partnership with those students who have had shared experiences, whilst useful, should be mindful not to place all the responsibilities for this on an over-burdened cohort of students.

Many medical students avoid seeking help, even more so when the university is involved due to the implications, they believe this will have on their futures as working professionals (27). This phenomenon in combination with the results above, highlights that whilst medical schools have specialist support systems in place to support students, the support that is desired from students is from their peers. Our study highlights the need for medical schools to implement programmes within the degree that help equip their students with the tools to best support their peers within their capacity. We have also highlighted the need for medical schools to help ensure there is a bridge between students across different year groups. This could be done through schemes such as mentoring pairings between older and younger years, ensuring a degree of compatibility between the mentor and mentee so that they have those shared or similar experiences.

Table 2 provides a summary of our findings and explores potential recommendations for medical schools to help improve the sense of belonging for students impacted by discrimination.

Table 2 A summary of the key findings of this study and a summary of recommendations for medical schools to help improve the sense of belonging experiences by students impacted by discrimination.

Findings	Recommendations for medical schools

For peer review only

Feelings of exclusion, imposter syndrome and low self-worth are common amongst students experiencing discrimination.	Workshops at the start of medical school to raise awareness of imposter syndrome and its prevalence within the medical school and the possible implementation of support for students, potentially through peer mentors and staff, for students who possess characteristics at risk of discrimination. We can perhaps further empower those who have these experiences to work in partnership with institutions in designing these workshops and where appropriate are rewarded for their time in remuneration or recognition.
Participants desire a sense of community to meet like-minded individuals.	Networking and peer social events for students to meet and discuss ideas and share experiences.
Feelings of inclusion can be fostered through representative teaching and the diversity observed amongst staff.	Ensure teaching considers the social and cultural diversity of the student body and ensure diversity amongst the student-facing workforce.

STRENGTHS AND LIMITATIONS

Previous research typically focuses on exploring belonging from a generic student perspective or from students belonging to a specific socio-demographic background. Instead, our research examined student belonging from a broader perspective, hearing from those impacted by discrimination, and acknowledging the multiple identities that students can have. This study utilised a novel method of exploring peer perspectives via a team largely composed of medical student researchers, whom themselves have lived experiences of discrimination within medical school. The student-led nature of this research allowed for a deeper and clearer understanding of student experiences. Our research not only contributes to the knowledge in this area by providing information on the personal experiences of medical students impacted by discrimination but importantly highlights areas for change and recommendations for support that could be offered by universities that may improve the sense of belonging for these students.

Nevertheless, this multi-institutional study covers only 4 of the 46 current medical schools in the UK. Therefore, further research is required to fully explore each medical school's support structure for medical students impacted by discrimination, such as further examining the experiences of students with intersecting identities and the experiences across varying geographical locations within the UK and beyond. Furthermore, the heterogeneous nature of medical programmes across institutions is something that must be accounted for when considering the experiences of belonging and implementations for change. We would like to further explore these recommendations by taking the findings of this research to the faculty leaders in these institutions and other medical school bodies to stimulate further discussion on what strategies they may implement to improve these students' sense of belonging.

For peer review only

CONCLUSION

In this study, we explored the experiences of the sense of belonging for medical students impacted by discrimination and the support systems they accessed. The findings of this study suggest medical schools are failing to recognise the importance of acknowledging the multiple identities held by students. Also, there is a lack of social and cultural awareness amongst peers and support structures that can often perpetuate feelings of isolation and exclusion. Furthermore, representation and the lack thereof can drive further feelings of lack of belonging and values of self-importance among students. However, positive interpersonal relationships among peers and those with similar lived experiences, alongside accessible, personalised support pathways, can provide emotional safety and support for students to feel more included.

Medical educators and institutions must consider inclusive, representative curricula and diversity amongst their student-facing staff. Furthermore, both formal and informal provisions should be offered for students to build communities and networks amongst peers to allow students impacted by discrimination to foster a greater sense of belonging.

Our research highlights the need for practical systemic initiatives to improve the sense of belonging of medical students who are impacted by discrimination, appreciating the multiple identities students may hold and how this influences the experiences they may have whilst at medical school.

Contributors

H Ikhlaq, S Agarwal, C Kwok, H Golamgouse, S Derby and N McRae contributed to the study conception, protocol development, data collection, data interpretation and critical revision of the manuscript. M Brown, V Collin, R Parekh and S Kumar provided supervising guidance and support utilising their expertise in the field of medical education.

Funding

This article presents independent research commissioned by the National Institute for Health Research (NIHR) under the Applied Health Research (ARC) programme for North West London. The views expressed in this publication are those of the author(s) and not necessarily those of the NHS, the NIHR or the Department of Health and Social Care.

Competing interests

The authors declare that they have no competing interests. The authors alone are responsible for the content and writing of the article.

Consent for publication

For peer review only

Authors have agreed to submit it in its current form for consideration for publication in the journal. No patient consent required.

Ethics approval and consent to participate
Ethical approval was given by Imperial College London, with reciprocal approval granted by partner medical schools (reference number: EERP2122-068a). Consent was gained prior to the interview through submission of an online consent form and verbal consent to record the session was obtained prior to the start of each discussion.

Data availability statement
Not applicable.

Notes on contributors

Hamza Ikhlaq is the lead researcher and a 5th year medical student at Imperial College London.

Srishti Agarwal is a 5th year medical student at University College London.

Catherine Kwok is a 5th year medical student at University College London.

Hadiya Golamgouse is a 5th year medical student at University College London.

Simone Derby is a 4th year medical student at the University of Nottingham Medical Schools.

Nicola McRae is a 3rd year medical student at the University of Dundee.

Dr Megan Brown is a Teaching Fellow in Medical Education Research with the Medical Education Innovation and Research Centre (MEdIC) at Imperial College London.

Dr Victoria Collin is a Teaching Fellow in Medical Education Research with the Medical Education Innovation and Research Centre (MEdIC) at Imperial College London.

Dr Ravi Parekh is the Director of Medical Education Innovation and Research at Imperial College London.

Professor Sonia Kumar is a Professor of Medical Education and Associate Dean for Community-Engaged Learning at the University of Leeds.

REFERENCES

1. APA Dictionary of Psychology. 2023. Discrimination.
2. Strayhorn TL. College students' sense of belonging. New York, NY. Routledge 2018. doi: 10.4324/9781315297293
3. Pittman L, Richmond A. University belonging, friendship quality, and psychological adjustment during the transition to college. *J Exp Educ* 2008;76(4):343-362. doi: 10.3200/JEXE.76.4.343-362

For peer review only

4. Jochman J, Cheadle J, Goosby B, et al. Mental health outcomes of discrimination among college students on a predominantly White campus; a prospective study. *Socius* 2019;5. doi: 10.1177/2378023119842728
5. Nguyen M, Chaudhry SI, Desai MM, et al. Association of mistreatment and discrimination with medical school attrition. *JAMA Pediatr* 2022;176(9):935-937. doi: 10.1001/jamapediatrics.2022.1637
6. (HEFCE) Higher Education Funding Council for England, (OFFA) Office for Fair Access. National strategy for access and student success in higher education. In: Department of Business Innovation & Skills, editor. 2014. https://assets.publishing.service.gov.uk/government/uploads/system/uploads/attachment_data/file/299689/bis-14-516-national-strategy-for-access-and-student-success.pdf
7. (BMA) British Medical Association. Widening participation in medicine. [Online]. 2023. [Available from: https://www.bma.org.uk/advice-and-support/studying-medicine/becoming-a-doctor/widening-participation-in-medicine]
8. Srinivas J, Panda A, Ferguson A, et al. A review of differential attainment in recruitment in the UK medical profession: Bridging the gap – thematic series on tackling differential attainment in medical profession 20-21. *Sushruta J Health Pol* 2021;14(1):1-15. doi: 10.38192/14.1.12
9. Jackson L. Response to When I say…fairness in selection. *Medical Education* 2018;53. doi: 10.1111/medu.13741
10. Krstic C, Krstic L, Tulloch A, et al. The experience of widening participation students in undergraduate medical education in the UK: A qualitative systematic review. *Medical Teacher* 2021;43(9):1044-1053. doi: 10.1080/0142159X.2021.1908976
11. Fyfe M, Horsburgh J, Blitz J, et al. The do's, don'ts and don't knows of redressing differential attainment related to race/ethnicity in medical schools. *Perspect Med Educ* 2021;11(1):1-14. doi: 10.1007/s40037-021-00696-3
12. Bettencourt G. "I belong because it wasn't made for me": understanding working-class students' sense of belonging on campus. *J Higher Educ* 2021;92(5):760-783. doi: 10.1080/00221546.2021.1872288
13. Morrison N, Machado M, Blackburn C. Student perspectives on barriers to performance for black and minority ethnic graduate-entry medical students: a qualitative study in a West Midlands medical school. *BMJ Open* 2019;9(11):e032493. doi: 10.1136/bmjopen-2019-032493
14. Shaw SC, Anderson JL, Grant AJ. Studying medicine with dyslexia: a collaborative autoethnography. *Qual Rep* 2016;21(11):2036-2054. doi: 10.46743/2160-3715/2016.2570
15. Broad J, Matheson M, Verrall F, et al. Discrimination, harassment and non-reporting in UK medical education. *Med Educ* 2018;52(2):414-426. doi: 10.1111/medu.13529
16. Braun V, Clarke V. Using thematic analysis in psychology. *Qual Res Psychol* 2006;3(2):77-101. doi: 10.1191/1478088706qp063oa
17. Thorne S, Kirkham S, MacDonald-Emes J. Interpretive description: a noncategorical qualitative alternative for developing nursing knowledge. *Res Nurs Health* 1997;20(2):169-177. doi: 10.1002/(sici)1098-240x(199704)20:2<169::aid-nur9>3.0.co;2-i
18. Connel-Smith A, Hubble S. Widening participation strategy in higher education in England. In: House of Commons Library, editor. 2018.

For peer review only

19. Whitla D, Orfield G, Silen W, et al. Education benefits of diversity in medical school: a survey of students. *Acad Med* 2003;78(5):460-466. doi: 10.1097/00001888-200305000-00007
20. Machado M, Ribeiro D, de Carvalho Filho M. Social justice in medical education: inclusion is not enough – it's just the first step. *Perspect Med Educ* 2022;11(4):187-195. doi: 10.1007/s40037-022-00715-x
21. Salehi PP, Jacobs D, Suhail-Sindhu T, et al. Consequences of medical hierarchy on medical students, residents, and medical education in Otolaryngology. *Otolaryngol Head Neck Surg* 2020;163(5):906-914. doi: 10.1177/0194599820926105
22. Bedi S. Increasing knowledge and inclusivity – encompassing BAME in medical education. *Med Teach* 2021;43(8):974-975. doi: 10.1080/0142159X.2020.1817350
23. Vogan C, McKimm J, Da Silva A, et al. Twelve tips for providing effective student support in undergraduate medical education. *Med Teach* 2014;36(6):480-485. doi: 10.3109/0142159X.2014.907488
24. van Buuren A, Yaseen W, Veinot P, et al. Later is too late: exploring student experiences of diversity and inclusion in medical school orientation. *Med Teach* 2021;43(5)-538-545. doi: 1080.0142159X.2021.1874326
25. Abrams M, Salzman J, Rey A, et al. Impact of providing peer support on medical students' empathy, self-efficacy, and mental health stigma. *Int J Environ Res Public Health* 2022;19(9):5135. doi: 10.3390/ijerph19095135
26. Graves J, Flynn E, Woodward-Kron R, et al. Supporting medical students to support peers: a qualitative interview study. *BMC Med Educ* 2022;20(1). doi: 10.1186/s12909-022-03368-w
27. Shafan-Oren B, Madan I, Henderson C. "A lot of medical students, their biggest fear is failing at being seen to be a functional human": disclosure and help-seeking decisions by medical students with health problems. *BMC Med Educ* 2021;21(1) doi: 10.1186/s12909-021-03032-9

For peer review only

APPENDICES

Appendix 1 - Interview Questions

1. Warm up: Can you tell me how you have generally found studying medicine? Is there any particular reason why you chose this medical school?
Prompt: Confidence with learning, and workload levels?

2. Have you had any experiences during your time at medical school that made you feel as though you belonged?

Prompt: Could you tell me a little bit more about that? What did you learn from that experience? How has this experience impacted you going forward?

3. Have you had any experiences during your time at medical school that made you feel as though you didn't belong?

Prompt: Could you tell me a little bit more about that? What did you learn from that experience? How has this experience impacted you going forward?

4. Have you found anything to be particularly challenging or difficult as a medical student impacted by discrimination?
Prompt: Can you tell me a bit more about that? What did you learn from that experience? Will/how will that change how you see things or act moving forwards?

5. What support currently exists for you at medical school? Have you accessed any of these? Has this proved satisfactory/useful?
Prompt: Are there any structures in particular? How many of these systems are student-led?

6. What support currently exists for you outside of the formal support provided by your medical school?
Prompt: Does the support within medical school or these wider networks foster a greater sense of belonging?

7. At the moment, how are your working relationships within your medical school? (Rephrase if not understood - How do you feel like your professional relationships with your peers are? And then can ask about medical school staff).
Prompt: And what about your peers? What about with staff at the medical school?

8. How are your working relationships on clinical placements? (Rephrase if not understood - How do you feel like your professional relationships with the staff on placements are?)

Prompt: With doctors? And with patients?

— TO ASK TO ROUND UP Q8: Do you feel like you belong in the clinical environment?

9. At the moment, are there any areas where you feel you could do with more support/supervision to improve your sense of belonging?
Prompt: Do you feel there is anything lacking in terms of support at your medical school? And in what areas do you feel you are represented in the curriculum? Can this be improved?

10. At the moment, how has the experience of being discriminated against impacted your overall feelings regarding your studies at medical school?
Prompt: How has this impacted your sense of belonging in particular?
Has anything changed for you over your time at university in terms of feeling a sense of belonging?
How has this impacted your feelings about becoming a doctor and being a part of the medical profession?

11. Is there anything else you'd like to share about your experiences of belonging and support at medical school?
Prompt: Is there anything you feel we haven't covered in this interview that we should have?

For peer review only

12. Do you have any questions that you would like to ask?

Debriefing information (see welfare support sheet)

Appendix 2 - Recruitment Text

Medical students impacted by discrimination: Their experiences of sense of belonging and support systems at medical school.

With so many initiatives to improve the diversity of medical schools, we're a team of UK medical students interested in finding out just how much minoritized medical students feel they belong and are supported past the application process of their respective schools. We are interested in hearing your honest experiences in a bid to help shape medical education in the UK.

To participate in this study you must have attended or are currently attending the following medical schools:

- Imperial College London
- University College London
- University of Dundee
- University of Nottingham Schools (Nottingham and Lincoln Campus)

For the purposes of this research, we have defined minoritized as someone who has felt that they have been impacted by discrimination. Examples of minoritized groups include (but are not limited to) the following:

- Those who identify as being otherly-abled/ having a disability/ Receiving DSA
- Those who belong to an ethnic minority
- Those who identify as LGBTQIA+
- First generation applicants to higher education
- Those previously looked after by a local authority
- Those who are themselves, or have parents/ guardians in receipt of means-tested benefits
- Household addresses in a POLAR 3 or 4 area
- Those with refugee status
- A young carer
- Students who belong to one of the priority engagements groups (include students from Gypsy, Roma and Traveller communities and children on military families)
- Those who are estranged from both parents/ legal guardians during their higher education
- Mature students

All students who apply and are selected to participate in our interviews, which should take no longer than 60 minutes, will receive a £20 shopping voucher at the end of the process as compensation for their time.

For peer review only

If you are interested in taking part, please click the link to the following survey to register your interest:

tinyurl.com/ICLSEDFORM

If you have any questions regarding this project, please contact: Hamza Ikhlaq via email: hi418@ic.ac.uk

For peer review only

BMJ Open

Medical students impacted by discrimination: a qualitative study into their experiences of belonging and support systems at medical schools in the United Kingdom

Journal:	*BMJ Open*
Manuscript ID	bmjopen-2023-078314.R1
Article Type:	Original research
Date Submitted by the Author:	27-Nov-2023
Complete List of Authors:	Ikhlaq, Hamza; Imperial College London, Faculty of Medicine<br>Agarwal, Srishti; University College London<br>Kwok, Catharine; University College London<br>Golamgouse, Hadiya; University College London<br>Derby, Simone ; University of Nottingham<br>McRae, Nicola; University of Dundee<br>Brown, Megan; Imperial College London, Medical Education Innovation and Research Centre (MEdIC)<br>Collin, Victoria; Imperial College London, Medical Education Innovation and Research Centre (MEdIC)<br>Parekh, Ravi; Imperial College London, Medical Education Innovation & Research Centre, School of Public Health<br>Kumar, Sonia; University of Leeds
**Primary Subject Heading**:	Medical education and training
Secondary Subject Heading:	Qualitative research
Keywords:	MEDICAL EDUCATION & TRAINING, QUALITATIVE RESEARCH, Social Support

SCHOLARONE™
Manuscripts

I, the Submitting Author has the right to grant and does grant on behalf of all authors of the Work (as defined in the below author licence), an exclusive licence and/or a non-exclusive licence for contributions from authors who are: i) UK Crown employees; ii) where BMJ has agreed a CC-BY licence shall apply, and/or iii) in accordance with the terms applicable for US Federal Government officers or employees acting as part of their official duties; on a worldwide, perpetual, irrevocable, royalty-free basis to BMJ Publishing Group Ltd ("BMJ") its licensees and where the relevant Journal is co-owned by BMJ to the co-owners of the Journal, to publish the Work in this journal and any other BMJ products and to exploit all rights, as set out in our licence.

The Submitting Author accepts and understands that any supply made under these terms is made by BMJ to the Submitting Author unless you are acting as an employee on behalf of your employer or a postgraduate student of an affiliated institution which is paying any applicable article publishing charge ("APC") for Open Access articles. Where the Submitting Author wishes to make the Work available on an Open Access basis (and intends to pay the relevant APC), the terms of reuse of such Open Access shall be governed by a Creative Commons licence – details of these licences and which Creative Commons licence will apply to this Work are set out in our licence referred to above.

Other than as permitted in any relevant BMJ Author's Self Archiving Policies, I confirm this Work has not been accepted for publication elsewhere, is not being considered for publication elsewhere and does not duplicate material already published. I confirm all authors consent to publication of this Work and authorise the granting of this licence.

For peer review only

Medical students impacted by discrimination: a qualitative study into their experiences of belonging and support systems at medical schools in the United Kingdom

Hamza Ikhlaq[1], Srishti Agarwal[2], Catharine Kwok[2], Hadiya Golamgouse[2], Simone Derby[3], Nicola McRae[4], Dr Megan Brown[1], Dr Victoria Collin[1], Dr Ravi Parekh[1] and Professor Sonia Kumar[5]

[1]Imperial College London, The Reynolds Building,
St Dunstans Road, London, W6 8RP

[2]University College London,
74 Huntley Street, London, WC1E 6BT

[3]University of Nottingham Medical Schools
East Block, Lenton, Nottingham NG7 2UH

[4]University of Dundee,
Nethergate, Dundee, DD1 4HN

[5]University of Leeds,
Faculty of Medicine and Health, Leeds, LS2 9JT

Correspondence concerning this article should be addressed to Hamza Ikhlaq
Imperial College London, The Reynolds Building
St Dunstans Road, London, W6 8RP
Email: hamza.ikhlaq18@imperial.ac.uk

Abstract: 198 words
Manuscript: 4312 words

For peer review only

ABSTRACT

Objective: To better understand the broader experience of medical students impacted by discrimination and the support systems they engage with.

Design: Qualitative study using semi-structured interviews.

Setting: Four medical schools based in the United Kingdom.

Participants: 17 medical students were recruited using volunteer and snowball sampling: all students self-identified as being impacted by discrimination.

Results: 5 themes were identified: feelings of isolation, imposter syndrome and exclusion; a lack of representation and positive role modelling; the importance of peer support; issues relating to the accessibility of support; building support networks through shared experiences and attempts to foster a sense of inclusion through peer and institutionally led initiatives.

Conclusions: The findings of this study suggest medical schools could do more to recognise the importance of acknowledging the multiple identities at risk of discrimination held by students, perpetuating feelings of isolation and exclusion. Our research highlights the need for practical systemic initiatives to improve the sense of belonging of medical students who are impacted by discrimination. Medical educators and institutions should consider formal and informal provisions, such as creating time and space for students to meet and share experiences, access support and reporting networks, to foster a greater sense of belonging.

Strengths and limitations of this study

- This study utilised a novel method of exploring peer perspectives via a team largely composed of medical student researchers, whom themselves have lived experiences of discrimination within medical school.
- Our research is unique in its approach as it examines student belonging from a broader perspective, hearing from those impacted by discrimination, and acknowledging the multiple identities that students can have.
- Although this study has a small sample, it captures the range of experiences across four institutions and across multiple years of medical school programmes.
- Given the complexity and sensitivity of discrimination in medical school, semi-structured interviews have allowed us to explore the personal and often sensitive experiences of participants.
- This multi-institutional study covers 4 of the 46 current medical schools in the UK. Therefore, there is scope for further examining the experiences of students with intersecting identities and experiences across varying geographical locations within the UK and beyond.

For peer review only

Funding statement

This article presents independent research commissioned by the National Institute for Health Research (NIHR) under the Applied Health Research (ARC) programme for North West London. The views expressed in this publication are those of the author(s) and not necessarily those of the NHS, the NIHR or the Department of Health and Social Care.

Keywords

discrimination, belonging, medical, student, support

INTRODUCTION

To experience discrimination is to be treated unfairly or with prejudice based on the characteristics of a group, or an individual (1). Terrell L. Strayhorn (2) defined a sense of belonging within higher education as:

> 'students' perceived social support on campus, a feeling or sensation of connectedness, and the experience of mattering or feeling cared about, accepted, respected, valued by, and important to the campus community or others on campus such as faculty, staff, and peers.' (p.4).

Belonging has been linked to positive academic outcomes, high engagement and self-confidence amongst university students (3) and is pertinent in students who are impacted by discrimination as they are already susceptible to exclusion and isolation (4). Such students who have reported mistreatment and discrimination in medical school, particularly those in their earlier years, have subsequently been found to be more likely to discontinue from medical school (5).

Historically, the makeup of medical students in the UK is skewed towards students from more affluent backgrounds (6). As a result, UK medical schools are now obliged to have schemes that ensure medicine is accessible to a wider population (7). Widening participation (WP) is an example of such an approach and is defined as: 'ensuring that students from disadvantaged backgrounds can access higher education, get the support they need to succeed in their studies, and progress to further study and/or employment suited to their qualifications and potential' (6). However, students impacted by discrimination , whether it be due to their WP status or those at risk of societal discrimination due to minoritised characteristics, have been found to face barriers in every stage of their medical careers (8).

According to Jackson, 'Fairness and equality in medical education is not just a problem at the front door; it continues throughout medical school.' (9) Current literature primarily focuses on interventions for post-16 students to boost enrolment into medical school. However, there is limited research into the follow up of students impacted by discrimination during their time in medical school (10). There is emerging evidence that students facing discrimination have different learning and social experiences and further research is required to address systemic issues within medical schools (11). Indeed, the prescriptive nature of simply increasing the

For peer review only

number of students from disadvantaged backgrounds into medical schools has a risk of 'tokenising' these students, and further alienating their sense of belonging (12). Medical students subject to discrimination can experience social exclusion from the wider cohort and inequality in accessing resources. This can result in a diminished sense of trust between these students and the institution (13). There are also specific barriers such as fears of stigmatisation and a lack of accessibility for neurodivergent students (14, 15), which influences a poorer sense of belonging due to poor relationships with the institution, staff, and clinicians (13). Though important, and interesting, these studies are limited in that they typically focus on exploring one group of student's sense of belonging such as ethnically minoritised students, or socio-economically disadvantaged populations.

There is a need for further research to examine the wider experiences of medical students impacted by discrimination and the intersections of identities for students who may have two or more characteristics that are at risk of discrimination. Furthermore, it is imperative to understand what support these students would require from their universities to maximise student engagement. Therefore, this project examined the experiences of medical students who have been impacted by discrimination, and the support systems available to them. We aim to generate practical recommendations to improve systemic and holistic provisions for these students thereby enhancing their sense of belonging.

METHODS

Given the complexity and sensitivity of discrimination in medical school, we chose to conduct semi-structured interviews to explore the thoughts, beliefs, and experiences of participants. We created 12 questions to form the basis of our semi-structured interviews covering various themes of belonging, networks, support systems and experiences of discrimination using findings from our literature review. Each participant was interviewed individually (Appendix 1). We used a constructivist paradigm (16) using an interpretative description approach (17). This enabled us to fully explore each participant's subjective experiences and understand the influences behind these, in order to develop practical recommendations to improve belonging and support systems at medical school. Data were collected between October to December 2022; ethical approval was given by the Educational Ethics Research Committee at **Imperial College London,** with reciprocal approval granted by partner medical schools of the research team (reference number: EERP2122-068a). All participants were emailed welfare debriefing information specific to their institution and SK was the assigned point of contact in the event of serious concern.

Sample and Recruitment

Medical students who self-identified as having been impacted by discrimination were eligible to take part in the study. Students who had just undertaken an intercalated BSc were excluded from the study, as they may be affiliated to a department external to the medical school and not all medical students may have the opportunity to intercalate. Participants self-identified as having been impacted by discrimination using an existing, broadened widening participation definition (18). This allowed us to collect a diverse multitude of student experiences, which may be currently underrepresented in existing literature and policies. Our recruitment text provided some guidance through examples of discriminated groups including but not limited to:

For peer review only

- Those who identify as being otherly-abled/having a disability/receiving DSA
- Those who belong to an ethnic minority
- Those who identify as LGBTQIA+
- First generation applicants to higher education
- Those previously looked after by a local authority
- Those who are themselves, or have parents/guardians in receipt of means-tested bene benefits
- Household addresses in a POLAR 3 or 4 area
- Those with refugee status
- A young carer
- Students who belong to one of the priority engagements groups (include students from Gypsy, Roma and Traveller communities and children on military families)
- Those who are estranged from both parents/legal guardians during their higher education
- Mature students

For the full recruitment text, see Appendix 2.

We recruited through the host institution using email, newsletters, and relevant social media platforms. Adverts were sent out through these platforms asking students to sign up. These students were then contacted by the research team to check eligibility and availability for an interview.

In addition to fulfilling our recruitment criteria, participants were eligible to participate if they were available for an interview and provided consent to participate. We aimed to ensure breadth of experiences, maximising variability across year groups; for each university, we selected one student per year group. All participants were compensated for their time through £20 food vouchers. In total, we recruited 17 participants from all years of the medical school programmes across four UK-based medical schools using convenience sampling. This allowed us to achieve our study aims2 and ensure a breadth of experiences, exploring their experiences to draw conclusions regarding their sense of belonging and experience of support at medical school.

Procedure

For peer review only

After selection, each participant was invited to a 60-minute semi-structured interview. Each interview was run by two co-investigators (HI, SA, CK, HG, NM, SD), with one leading and one supporting the interview (prompting additional questions to explore students' experiences or in case of technical difficulties). Consent was gained prior to the interview through submission of an online consent form and verbal consent to record the session was obtained prior to the start of each discussion. To avoid potential conflicts of interest, investigators only interviewed participants external to their own institutions.

Data Analysis

After transcription, we anonymised data and followed a constructivist approach to qualitative thematic analysis. We analysed data inductively and followed Braun and Clarke's (16) six steps: familiarisation; data coding; generating initial themes; reviewing and developing themes; refining, defining and naming themes; and writing the report. We developed a code list, which all members of our team reviewed. At least one member of our team peer-reviewed each transcript to add depth to coding (HI, SA, CK, HG, NM, SD). Throughout the analysis, we modified existing codes and created new codes through discussion. We then connected these codes to develop themes through team discussions. All data analysis was conducted using the Dedoose (v9.0) software.

Patient and public involvement

Patients or the public were not involved in the design or conduct of this study.

Reflexivity

The research team come from varying ethnically minoritised backgrounds and is predominantly female identifying, all with a strong interest in medical education. All student researchers identify as having been impacted by discrimination, thus leading the research team to explore this research project and have a strong aspiration to make positive recommendations for medical schools. Due to the nature of our study and personal experiences of discrimination, peer debriefing through online meetings was used to reflect and deepen our understanding of recounted experiences shared by study participants and provide support if sensitive topics arose.

RESULTS

Five themes were identified from the 17 interviews conducted: feelings of exclusion; failures of organised support systems; representation within the medical school curriculum, staff and peers; the importance of peer support and attempts to foster a sense of inclusion. A summary of the characteristics of our study population is provided below (Table 1).

Table 1 Characteristics of our Study Population

For peer review only

Participant	Gender	Medical School	Year Group in the academic year 2021-2022
P001	Male	Imperial College London	1
P002	Non-Binary	Imperial College London	2
P003	Male	Imperial College London	3
P004	Non-Binary	Imperial College London	4
P005	Female	Imperial College London	5
P006	Female	University College London	1
P007	Female	University College London	2
P008	Male	University College London	3
P008	Female	University College London	4
P009	Male	University of Nottingham and Lincoln Medical Schools	1
P010	Female	University of Nottingham and Lincoln Medical Schools	2
P011	Female	University of Nottingham and Lincoln Medical Schools	3
P012	Female	University of Nottingham and Lincoln Medical Schools	4
P013	Female	University of Dundee	1
P015	Female	University of Dundee	2
P016	Female	University of Dundee	3
P017	Female	University of Dundee	4

For peer review only

Feelings of exclusion

One of the main barriers to achieving a sense of belonging experienced by participants was feelings of exclusion at an individual and institutional level. At an individual level, minoritised characteristics such as ethnicity, socioeconomic class, religious beliefs, and caring responsibilities were key factors which participants felt perpetuated feelings of exclusion. For example, one participant highlighted the lack of diversity amongst peers as the

reason they 'never feel' like they 'fully belong' (P012). This was further exacerbated by intersections of identity as 'being an ethnic minority wearing a hijab' made them feel they were 'a minority within a minority' (P012). Opportunities to socialise with peers sometimes conflicted with participants' cultural and religious beliefs where 'every single event centred around drinking' with a lack of alternative events available (P005). Furthermore, participants highlighted when feelings of exclusion were felt it was because they 'couldn't connect' to their peers due to their unique experiences such as 'experiences as a care leaver', thus resulting in self 'blame' to explain the 'disconnect' (P002).

At an institutional level, participants expressed a disconnect between the medical schools and students. One student felt that they felt they were 'set up to fail' in a system which does not recognise their intersection of identities, thus 'disempowering' them (P008). Feelings of exclusion echoed into clinical attachments where students felt senior doctors would make 'split-second' judgements based on 'prejudice', to the extent they felt 'belittled' (P012). Examples of this emerged particularly in surgical theatres where students who wore hijab felt 'discouraged from going to theatre' (P012).

Failures of Support Systems

Participants had experienced failings in the formal support networks and structures provided by their medical schools. Fixed structures, such as requiring a tutor referral for welfare support made it 'very difficult… to access other welfare support services' (P002) when their tutor had been uncontactable for an extended period of time. This barrier to accessing pastoral support was further demonstrated by another participant questioning: 'how desperate are you to go through all… those hoops to actually get the support?' (P004).

Other failings have included experiences of being 'bounced between people and no one really is quite sure what's happening' (P008). Consequently, this 'one size fits all' (P003) approach has built a feeling of lack of genuine care: 'they want to tick a box saying they've provifded support' (P012) and one participant expressed that they had 'a level of distrust …within the system' (P008). Lack of follow-up and ongoing support was evidenced: 'it almost feels prescriptive, that's just like okay, we've met, great, we've done our job, you must be better now' (P004).

Representation

Representation in medical school was a common topic that was raised as a source of comfort, helping to improve belonging, and equally, the lack thereof challenged participants.

Having an open space to discuss identities, alongside others similarly identifying individuals helped provide a sense of familiarity to participants, namely for LGBTQ+ individuals who struggled to find this in the 'heteronormative blanket' (P008). Additionally, the diversity of faculty members also greatly helps to improve belonging, with one participant 'so genuinely happy to see a hijabi lecturer because it was the only one I've seen' (P012).

Issues with representation arose on several occasions, such as the integral makeup of students with 'most' of the student population being educated in 'private schools' and the class divide that inherently exists between these students and students impacted by discrimination (P012). Furthermore, participants felt they may be represented in faculty as a 'woman' but did not

For peer review only

feel the same with regards to their 'ethnicity'. (P013) Additionally, the lack of inclusive teaching, including signs and skin pathology in ethnic minority patients, were raised twice (P012, P013) as a significant problem that impacts belonging. Indeed, the '(lack of teaching) makes you feel like *you* don't matter, *you're not important*.' (P012)

Despite this, one student did raise some improvements that have been made as a whole, which has improved their sense of belonging through representation. There has been more 'active efforts to embrace people of different backgrounds' in clinical and academic environments which have been 'successful' and have increased their sense of belonging (P003).

Peer Support

Some participants described how peer support, resulting from positive interpersonal relationships between themselves and members of their cohort, and the wider university population have impacted their experiences at medical school as students who have experienced discrimination. For those who have very little or no peer support, there was a desire to have that access. Having friends to help provide support was said to be integral in providing 'emotional padding' (P002) which some students have found was often omitted from their lives up to this point. To some students, the willingness of others to talk 'openly and give support' is a 'big deal' (P003), especially for those who are going through what seems to be challenging times.

However, participants specified preferences that would help maximise the efficiency of peers as a support system which included peers who are 'of older years' P002 and/ or with whom they 'have that shared experience' (P001). This stems from the notion that as much as 'peers who aren't from that background try to empathise and try to relate…they don't have the lived experience' (P003) which creates a barrier in feeling understood and supported.

Feelings of Inclusion

Participants tended to express feelings of inclusion when reflecting on their experiences with communities that they 'had a lot in common with' (P002). The freedom to be who you are without judgement was important to most students who found comfort in diverse student groups ranging from sports to LGTBQ+ societies. Feeling like 'something bigger than yourself' gave students the strength to 'be sort of whatever you want to be' (P003) and which in turn improved their overall sense of belonging.

However, feeling included was a process that required active effort 'I've had to fight to think that I belong' (P003). Several participants changed elements of themselves to better fit in, which only added to feeling more out of place. Feelings of inclusion were also constantly challenged; by themselves, by their peers, and by the medical school throughout their time at university. Imposter syndrome was attributed to this challenge. Students struggled to keep up with their peers and thought they weren't ''smart enough' (P008) to be at medical school. The lack of support at this level meant that students held onto these beliefs until they found a community which gave them 'the confidence to do well, but also equally fail' (P003).

For peer review only

Additionally, several students considered loneliness when reflecting on their experiences of inclusion. Many described feeling isolated from their peers due to characteristics largely outside of their control such as gender, and sexuality. Despite overcoming several hurdles to reach medical school, participants described feeling as if they alone did not belong and that they were out of place. As stated by P002 'For me, I took most of my first year of medical school thinking 'oh my god, I'm the only one' that everyone else was normal'. The importance of community was therefore highlighted in all participant reflections and was explored as a primary cause for improved feelings of inclusion.

DISCUSSION

Through semi-structured interviews, we explored the perceived sense of belonging felt by medical students across four UK medical schools who have been impacted by discrimination and the support systems they have accessed. Our study highlights how feelings of exclusion impact medical students' experiences at medical school. Students from minoritised backgrounds reported a lack of peer diversity and understanding of their experiences from peers. Furthermore, barriers to engaging in accessible social and professional activities often impacted participants' self-esteem.

Diversity in student makeup has been found to be associated with perceived enhancements in educational experiences (19). Furthermore, works by Machado et al. have suggested socioeconomic and racial diversity offers opportunities to reform medical culture, but student socialisation must be supported through activities to support self-esteem (20). Students also described experiences of marginalisation and stereotyping in clinical attachments, exacerbated by the professional hierarchy, which has been linked to moral distress, trainee mistreatment and amplification of fatigue, stress, and burnout (21). As well as this being reflected in our study, we find feelings of self-blame and a resultant lack of self-worth sometimes are used by students to explain the disconnect between their experiences and those of their non-marginalised peers, further disempowering them from fully engaging with clinical, non-clinical and social experiences provided within the medical school.

The need for representation extends within the formal curricula as participants highlighted the lack of inclusive teaching on disease in ethnically minoritised patients. Considering the topics discussed above, it is perhaps not surprising that participants felt displaced when teaching solely focused on white skin, with signs and symptoms only noted in those belonging to said populations (22). Participants in this study highlighted how seeing themselves represented within the curriculum allowed them to feel a greater sense of self-importance and observing active institutional efforts to diversify the curriculum fostered positive feelings towards their respective medical schools. Thus, representation, both in the physical form of a diverse and intersectional cohort of staff and doctors, coupled with inclusive, non-stereotyped, teaching, and the provision of allocated spaces to discuss these pertinent issues would greatly improve the sense of belonging for students impacted by discrimination.

Students reported experiences of barriers to accessing support through often hard-to-reach, unavailable networks, or outdated systems. The ease of access is a key area for institutions to focus on, for example, by providing multiple access points (23). Institutions should be proactively encouraging students to seek support at an earlier and more manageable stage, ensuring that follow-up opportunities are integrated. We have demonstrated that when a 'one

For peer review only

size fits all' and tokenistic system of support disappoints an individual, students have felt isolated and cynical. Providing individualised support tailored to appropriately match the diverse identities of the student population is paramount in order to bridge this disconnect between stated and perceived institutional values (24). We have demonstrated this is vital in ensuring students can build authentic connections with support staff to establish trust and reduce the confusion felt by students when attempting to access relevant support services. However, to implement such support, pastoral support needs to be prioritised and appropriately renumerated to overcome logistical barriers, such as time and financial costs.

This research has shown that across cohorts, medical students who have experienced discrimination desire peer-to-peer support, to help improve their sense of belonging. However, this peer support must be done in a way in which students feel will be most beneficial, such as from older students, those considered friends and those with a shared experience as them. Peer-to-peer support has been shown to help medical students who may feel burnt out, inadequate, socially isolated, and anxious (25), and is found to have positive impacts on relationships with peers as they become professionals (26). The development of peer support systems that are established in partnership with those students who have had shared experiences, whilst useful, should be mindful not to place all the responsibilities for this on an over-burdened cohort of students.

Many medical students avoid seeking help, even more so when the university is involved due to the implications, they believe this will have on their futures as working professionals (27). This phenomenon in combination with the results above, highlights that whilst medical schools have specialist support systems in place to support students, the support that is desired from students is from their peers. Our study highlights the need for medical schools to implement programmes within the degree that help equip their students with the tools to best support their peers within their capacity. We have also highlighted the need for medical schools to help ensure there is a bridge between students across different year groups. This could be done through schemes such as mentoring pairings between older and younger years, ensuring a degree of compatibility between the mentor and mentee so that they have those shared or similar experiences.

Table 2 provides a summary of our findings and explores potential recommendations for medical schools to help improve the sense of belonging for students impacted by discrimination.

Table 2 A summary of the key findings of this study and a summary of recommendations for medical schools to help improve the sense of belonging experiences by students impacted by discrimination.

Findings	Recommendations for medical schools
Feelings of exclusion, imposter syndrome and low self-worth are common amongst students experiencing discrimination.	Workshops at the start of medical school to raise awareness of imposter syndrome and its prevalence within the medical school and the possible implementation of support for students, potentially through peer mentors and staff, for students who possess characteristics at risk of discrimination. We can perhaps further empower those who

For peer review only

	have these experiences to work in partnership with institutions in designing these workshops and where appropriate are rewarded for their time in remuneration or recognition.
Participants desire a sense of community to meet like-minded individuals.	Networking and peer social events for students to meet and discuss ideas and share experiences.
Feelings of inclusion can be fostered through representative teaching and the diversity observed amongst staff.	Ensure teaching considers the social and cultural diversity of the student body and ensure diversity amongst the student-facing workforce.

STRENGTHS AND LIMITATIONS

Previous research typically focuses on exploring belonging from a generic student perspective or from students belonging to a specific socio-demographic background. Instead, our research examined students belonging from a broader perspective, hearing from those impacted by discrimination, and acknowledging the multiple identities that students can have. This study utilised a novel method of exploring peer perspectives via a team largely composed of medical student researchers, whom themselves have lived experiences of discrimination within medical school. The student-led nature of this research allowed for a deeper and clearer understanding of student experiences. Furthermore, our research was able to meet the criteria for conceptual depth by having a range of evidence and removing any need to make assumptions through the use of our in-depth semi-structured interviews (28).Our research not only contributes to the knowledge in this area by providing information on the personal experiences of medical students impacted by discrimination but importantly highlights areas for change and recommendations for support that could be offered by universities that may improve the sense of belonging for these students.

Nevertheless, this multi-institutional study covers only 4 of the 46 current medical schools in the UK. Therefore, further research is required to fully explore each medical school's support structure for medical students impacted by discrimination, such as further examining the experiences of students with intersecting identities and the experiences across varying geographical locations within the UK and beyond. There is the possibility of self-selection bias amongst the 17 participants; however, the self-identification inclusion criteria was broad and students were recruited across multiple year groups and medical schools. Although the cross-sectional design of this study yielded novel findings, further longitudinal studies are warranted to examine the long-term consequences for students impacted by discrimination, particularly as they progress throughout medical school and into their careers. Indeed, although beyond the scope of this paper, it would be interesting for future works to examine the differences in the sense of belonging felt outside of medical school compared to those experiences whilst in medical school. Furthermore, the heterogeneous nature of medical

For peer review only

programmes across institutions is something that must be accounted for when considering the experiences of belonging and implementations for change. We would like to further explore these recommendations by taking the findings of this research to the faculty leaders in these institutions and other medical school bodies to stimulate further discussion on what strategies they may implement to improve these students' sense of belonging.

CONCLUSION

In this study, we explored the experiences of medical students impacted by discrimination, their sense of belonging and the support systems they accessed. The findings of this study suggest positive interpersonal relationships among peers and those with similar lived experiences can provide emotional safety and support for students to feel more included. However, there remains a significant lack of social and cultural awareness amongst the student body and support structures that may perpetuate feelings of isolation and exclusion, persist. Furthermore, the lack of representation can drive feelings of a reduced sense of belonging and values of self-belief among students. To improve, medical educators and institutions must consider inclusive, representative curricula and diversity amongst their student-facing staff. Moreover, there is a need for medical schools to improve their support and recognition of the multiple identities held by students, through practical systemic initiatives. This includes, both formal and informal provisions offered to students to build communities and networks amongst peers, alongside accessible, personalised support pathways, thereby allowing students impacted by discrimination to foster a greater sense of belonging.

Contributors

H Ikhlaq, S Agarwal, C Kwok, H Golamgouse, S Derby and N McRae contributed to the study conception, protocol development, data collection, data interpretation and critical revision of the manuscript. M Brown, V Collin, R Parekh and S Kumar provided supervising guidance and support utilising their expertise in the field of medical education.

Funding

This article presents independent research commissioned by the National Institute for Health Research (NIHR) under the Applied Health Research (ARC) programme for North West London. The views expressed in this publication are those of the author(s) and not necessarily those of the NHS, the NIHR or the Department of Health and Social Care.

Competing interests

The authors declare that they have no competing interests. The authors alone are responsible for the content and writing of the article.

Consent for publication

For peer review only

Authors have agreed to submit it in its current form for consideration for publication in the journal. No patient consent required.

Ethics approval and consent to participate
Ethical approval was given by Imperial College London, with reciprocal approval granted by partner medical schools (reference number: EERP2122-068a). Consent was gained prior to the interview through submission of an online consent form and verbal consent to record the session was obtained prior to the start of each discussion.

Data availability statement
Not applicable.

Notes on contributors

Hamza Ikhlaq is the lead researcher and a final year medical student at Imperial College London.

Srishti Agarwal is a final year medical student at University College London.

Catharine Kwok is a final year medical student at University College London.

Hadiya Golamgouse is a final year medical student at University College London.

Simone Derby is a final year medical student at the University of Nottingham Medical Schools.

Nicola McRae is an intercalating medical student in BMSc Medical Education student at the University of Dundee.

Dr Megan Brown is a Teaching Fellow in Medical Education Research with the Medical Education Innovation and Research Centre (MEdIC) at Imperial College London.

Dr Victoria Collin is a Research Tutor/Lecturer in Psychology at the University of East London and a former Teaching Fellow at the Medical Education Innovation and Research Centre (MEdIC) at Imperial College London.

Dr Ravi Parekh is the Director of Medical Education Innovation and Research at Imperial College London.

Professor Sonia Kumar is a Professor of Medical Education and Associate Dean for Community-Engaged Learning at the University of Leeds.

REFERENCES

1. APA Dictionary of Psychology. 2023. Discrimination.
2. Strayhorn TL. College students' sense of belonging. New York, NY. Routledge 2018. doi: 10.4324/9781315297293

For peer review only

3. Pittman L, Richmond A. University belonging, friendship quality, and psychological adjustment during the transition to college. *J Exp Educ* 2008;76(4):343-362. doi: 10.3200/JEXE.76.4.343-362
4. Jochman J, Cheadle J, Goosby B, et al. Mental health outcomes of discrimination among college students on a predominantly White campus; a prospective study. *Socius* 2019;5. doi: 10.1177/2378023119842728
5. Nguyen M, Chaudhry SI, Desai MM, et al. Association of mistreatment and discrimination with medical school attrition. *JAMA Pediatr* 2022;176(9):935-937. doi: 10.1001/jamapediatrics.2022.1637
6. (HEFCE) Higher Education Funding Council for England, (OFFA) Office for Fair Access. National strategy for access and student success in higher education. In: Department of Business Innovation & Skills, editor. 2014. https://assets.publishing.service.gov.uk/government/uploads/system/uploads/attachment_data/file/299689/bis-14-516-national-strategy-for-access-and-student-success.pdf
7. (BMA) British Medical Association. Widening participation in medicine. [Online]. 2023. [Available from: https://www.bma.org.uk/advice-and-support/studying-medicine/becoming-a-doctor/widening-participation-in-medicine]
8. Srinivas J, Panda A, Ferguson A, et al. A review of differential attainment in recruitment in the UK medical profession: Bridging the gap – thematic series on tackling differential attainment in medical profession 20-21. *Sushruta J Health Pol* 2021;14(1):1-15. doi: 10.38192/14.1.12
9. Jackson L. Response to When I say…fairness in selection. *Medical Education* 2018;53. doi: 10.1111/medu.13741
10. Krstic C, Krstic L, Tulloch A, et al. The experience of widening participation students in undergraduate medical education in the UK: A qualitative systematic review. *Medical Teacher* 2021;43(9):1044-1053. doi: 10.1080/0142159X.2021.1908976
11. Fyfe M, Horsburgh J, Blitz J, et al. The do's, don'ts and don't knows of redressing differential attainment related to race/ethnicity in medical schools. *Perspect Med Educ* 2021;11(1):1-14. doi: 10.1007/s40037-021-00696-3
12. Bettencourt G. "I belong because it wasn't made for me": understanding working-class students' sense of belonging on campus. *J Higher Educ* 2021;92(5):760-783. doi: 10.1080/00221546.2021.1872288
13. Morrison N, Machado M, Blackburn C. Student perspectives on barriers to performance for black and minority ethnic graduate-entry medical students: a qualitative study in a West Midlands medical school. *BMJ Open* 2019;9(11):e032493. doi: 10.1136/bmjopen-2019-032493
14. Shaw SC, Anderson JL, Grant AJ. Studying medicine with dyslexia: a collaborative autoethnography. *Qual Rep* 2016;21(11):2036-2054. doi: 10.46743/2160-3715/2016.2570
15. Broad J, Matheson M, Verrall F, et al. Discrimination, harassment and non-reporting in UK medical education. *Med Educ* 2018;52(2):414-426. doi: 10.1111/medu.13529
16. Braun V, Clarke V. Using thematic analysis in psychology. *Qual Res Psychol* 2006;3(2):77-101. doi: 10.1191/1478088706qp063oa
17. Thorne S, Kirkham S, MacDonald-Emes J. Interpretive description: a noncategorical qualitative alternative for developing nursing knowledge. *Res*

Nurs Health 1997;20(2):169-177. doi: 10.1002/(sici)1098-240x(199704)20:2<169::aid-nur9>3.0.co;2-i
18. Connel-Smith A, Hubble S. Widening participation strategy in higher education in England. In: House of Commons Library, editor. 2018.
19. Whitla D, Orfield G, Silen W, et al. Education benefits of diversity in medical school: a survey of students. *Acad Med* 2003;78(5):460-466. doi: 10.1097/00001888-200305000-00007
20. Machado M, Ribeiro D, de Carvalho Filho M. Social justice in medical education: inclusion is not enough – it's just the first step. *Perspect Med Educ* 2022;11(4):187-195. doi: 10.1007/s40037-022-00715-x
21. Salehi PP, Jacobs D, Suhail-Sindhu T, et al. Consequences of medical hierarchy on medical students, residents, and medical education in Otolaryngology. *Otolaryngol Head Neck Surg* 2020;163(5):906-914. doi: 10.1177/0194599820926105
22. Bedi S. Increasing knowledge and inclusivity – encompassing BAME in medical education. *Med Teach* 2021;43(8):974-975. doi: 10.1080/0142159X.2020.1817350
23. Vogan C, McKimm J, Da Silva A, et al. Twelve tips for providing effective student support in undergraduate medical education. *Med Teach* 2014;36(6):480-485. doi: 10.3109/0142159X.2014.907488
24. van Buuren A, Yaseen W, Veinot P, et al. Later is too late: exploring student experiences of diversity and inclusion in medical school orientation. *Med Teach* 2021;43(5)-538-545. doi: 1080.0142159X.2021.1874326
25. Abrams M, Salzman J, Rey A, et al. Impact of providing peer support on medical students' empathy, self-efficacy, and mental health stigma. *Int J Environ Res Public Health* 2022;19(9):5135. doi: 10.3390/ijerph19095135
26. Graves J, Flynn E, Woodward-Kron R, et al. Supporting medical students to support peers: a qualitative interview study. *BMC Med Educ* 2022;20(1). doi: 10.1186/s12909-022-03368-w
27. Shafan-Oren B, Madan I, Henderson C. "A lot of medical students, their biggest fear is failing at being seen to be a functional human": disclosure and help-seeking decisions by medical students with health problems. *BMC Med Educ* 2021;21(1) doi: 10.1186/s12909-021-03032-9
28. Nelson, J. Using conceptual depth criteria: addressing the challenge of reaching saturation in qualitative research. *Qualitative Research* 2017; 17(5):554-570. Doi: 10.1177/1468794116679873

APPENDICES

Appendix 1 - Interview Questions

1. Warm up: Can you tell me how you have generally found studying medicine? Is there any particular reason why you chose this medical school?
Prompt: Confidence with learning, and workload levels?

2. Have you had any experiences during your time at medical school that made you feel as though you belonged?

Prompt: Could you tell me a little bit more about that? What did you learn from that experience? How has this experience impacted you going forward?

3. Have you had any experiences during your time at medical school that made you feel as though you didn't belong?
Prompt: Could you tell me a little bit more about that? What did you learn from that experience? How has this experience impacted you going forward?

4. Have you found anything to be particularly challenging or difficult as a medical student impacted by discrimination?
Prompt: Can you tell me a bit more about that? What did you learn from that experience? Will/how will that change how you see things or act moving forwards?

5. What support currently exists for you at medical school? Have you accessed any of these? Has this proved satisfactory/useful?
Prompt: Are there any structures in particular? How many of these systems are student-led?

6. What support currently exists for you outside of the formal support provided by your medical school?
Prompt: Does the support within medical school or these wider networks foster a greater sense of belonging?

7. At the moment, how are your working relationships within your medical school? (Rephrase if not understood - How do you feel like your professional relationships with your peers are? And then can ask about medical school staff).
Prompt: And what about your peers? What about with staff at the medical school?

8. How are your working relationships on clinical placements? (Rephrase if not understood - How do you feel like your professional relationships with the staff on placements are?)

Prompt: With doctors? And with patients?

— TO ASK TO ROUND UP Q8: Do you feel like you belong in the clinical environment?

For peer review only

9. At the moment, are there any areas where you feel you could do with more support/supervision to improve your sense of belonging?
Prompt: Do you feel there is anything lacking in terms of support at your medical school?
And in what areas do you feel you are represented in the curriculum? Can this be improved?

10. At the moment, how has the experience of being discriminated against impacted your overall feelings regarding your studies at medical school?
Prompt: How has this impacted your sense of belonging in particular?
Has anything changed for you over your time at university in terms of feeling a sense of belonging?
How has this impacted your feelings about becoming a doctor and being a part of the medical profession?

11. Is there anything else you'd like to share about your experiences of belonging and support at medical school?
Prompt: Is there anything you feel we haven't covered in this interview that we should have?

12. Do you have any questions that you would like to ask?

Debriefing information (see welfare support sheet)

Appendix 2 - Recruitment Text

Medical students impacted by discrimination: Their experiences of sense of belonging and support systems at medical schools in the United Kingdom.

With so many initiatives to improve the diversity of medical schools, we're a team of UK medical students interested in finding out just how much minoritized medical students feel they belong and are supported past the application process of their respective schools. We are interested in hearing your honest experiences in a bid to help shape medical education in the UK.

To participate in this study you must have attended or are currently attending the following medical schools:

- Imperial College London
- University College London
- University of Dundee
- University of Nottingham Schools (Nottingham and Lincoln Campus)

For the purposes of this research, we have defined minoritized as someone who has felt that they have been impacted by discrimination. Examples of minoritized groups include (but are not limited to) the following:

- Those who identify as being otherly-abled/ having a disability/ Receiving DSA
- Those who belong to an ethnic minority
- Those who identify as LGBTQIA+

For peer review only

- First generation applicants to higher education
- Those previously looked after by a local authority
- Those who are themselves, or have parents/ guardians in receipt of means-tested benefits
- Household addresses in a POLAR 3 or 4 area
- Those with refugee status
- A young carer
- Students who belong to one of the priority engagements groups (include students from Gypsy, Roma and Traveller communities and children on military families)
- Those who are estranged from both parents/ legal guardians during their higher education
- Mature students

All students who apply and are selected to participate in our interviews, which should take no longer than 60 minutes, will receive a £20 shopping voucher at the end of the process as compensation for their time.

If you are interested in taking part, please click the link to the following survey to register your interest:

tinyurl.com/ICLSEDFORM

If you have any questions regarding this project, please contact: Hamza Ikhlaq via email: hi418@ic.ac.uk

For peer review only

Standards for Reporting Qualitative Research (SRQR)*

http://www.equator-network.org/reporting-guidelines/srqr/

Title and abstract

	Page/line no(s).
**Title** - Concise description of the nature and topic of the study Identifying the study as qualitative or indicating the approach (e.g., ethnography, grounded theory) or data collection methods (e.g., interview, focus group) is recommended	3
**Abstract** - Summary of key elements of the study using the abstract format of the intended publication; typically includes background, purpose, methods, results, and conclusions	4

Introduction

**Problem formulation** - Description and significance of the problem/phenomenon studied; review of relevant theory and empirical work; problem statement	5-6
**Purpose or research question** - Purpose of the study and specific objectives or questions	6

Methods

**Qualitative approach and research paradigm** - Qualitative approach (e.g., ethnography, grounded theory, case study, phenomenology, narrative research) and guiding theory if appropriate; identifying the research paradigm (e.g., postpositivist, constructivist/ interpretivist) is also recommended; rationale**	8
**Researcher characteristics and reflexivity** - Researchers' characteristics that may influence the research, including personal attributes, qualifications/experience, relationship with participants, assumptions, and/or presuppositions; potential or actual interaction between researchers' characteristics and the research questions, approach, methods, results, and/or transferability	8
**Context** - Setting/site and salient contextual factors; rationale**	6
**Sampling strategy** - How and why research participants, documents, or events were selected; criteria for deciding when no further sampling was necessary (e.g., sampling saturation); rationale**	7
**Ethical issues pertaining to human subjects** - Documentation of approval by an appropriate ethics review board and participant consent, or explanation for lack thereof; other confidentiality and data security issues	6
**Data collection methods** - Types of data collected; details of data collection procedures including (as appropriate) start and stop dates of data collection and analysis, iterative process, triangulation of sources/methods, and modification of procedures in response to evolving study findings; rationale**	6

For peer review only

For peer review only - http://bmjopen.bmj.com/site/about/guidelines.xhtml

**Data collection instruments and technologies** - Description of instruments (e.g., interview guides, questionnaires) and devices (e.g., audio recorders) used for data collection; if/how the instrument(s) changed over the course of the study	8, 19
**Units of study** - Number and relevant characteristics of participants, documents, or events included in the study; level of participation (could be reported in results)	9
**Data processing** - Methods for processing data prior to and during analysis, including transcription, data entry, data management and security, verification of data integrity, data coding, and anonymization/de-identification of excerpts	8
**Data analysis** - Process by which inferences, themes, etc., were identified and developed, including the researchers involved in data analysis; usually references a specific paradigm or approach; rationale**	8
**Techniques to enhance trustworthiness** - Techniques to enhance trustworthiness and credibility of data analysis (e.g., member checking, audit trail, triangulation); rationale**	8

Results/findings

**Synthesis and interpretation** - Main findings (e.g., interpretations, inferences, and themes); might include development of a theory or model, or integration with prior research or theory	9
**Links to empirical data** - Evidence (e.g., quotes, field notes, text excerpts, photographs) to substantiate analytic findings	10-12

Discussion

**Integration with prior work, implications, transferability, and contribution(s) to the field -** Short summary of main findings; explanation of how findings and conclusions connect to, support, elaborate on, or challenge conclusions of earlier scholarship; discussion of scope of application/generalizability; identification of unique contribution(s) to scholarship in a discipline or field	12-14
**Limitations** - Trustworthiness and limitations of findings	14

Other

**Conflicts of interest** - Potential sources of influence or perceived influence on study conduct and conclusions; how these were managed	1, 8
**Funding** - Sources of funding and other support; role of funders in data collection, interpretation, and reporting	5

*The authors created the SRQR by searching the literature to identify guidelines, reporting standards, and critical appraisal criteria for qualitative research; reviewing the reference lists of retrieved sources; and contacting experts to gain feedback. The SRQR aims to improve the transparency of all aspects of qualitative research by providing clear standards for reporting qualitative research.

For peer review only

For peer review only - http://bmjopen.bmj.com/site/about/guidelines.xhtml

**The rationale should briefly discuss the justification for choosing that theory, approach, method, or technique rather than other options available, the assumptions and limitations implicit in those choices, and how those choices influence study conclusions and transferability. As appropriate, the rationale for several items might be discussed together.

Reference:

O'Brien BC, Harris IB, Beckman TJ, Reed DA, Cook DA. **Standards for reporting qualitative research: a synthesis of recommendations.** *Academic Medicine*, Vol. 89, No. 9 / Sept 2014 DOI: 10.1097/ACM.0000000000000388

For peer review only

For peer review only - http://bmjopen.bmj.com/site/about/guidelines.xhtml
